# Reliability of Reagent Strips in Veterinary Medicine: A Critical Evaluation of pH and Urinary Density in Dogs using Statistical Modeling

**DOI:** 10.1111/vcp.70124

**Published:** 2026-08-03

**Authors:** Cleber F. M. Mansano, Julia M. C. Ostti, Renata R. Latorre, Beatrice I. Macente, Amanda P. Lemes, Valentina C. Rosa

**Affiliations:** ^1^ Programa de Pós‐Graduação em Ciências Ambientais Universidade Brasil Fernandópolis Brazil; ^2^ Programa de Pós‐Graduação em Produção Animal Universidade Brasil Descalvado Brazil; ^3^ Departamento de Medicina Veterinária Universidade Brasil Fernandópolis Brazil; ^4^ Programa de Residência em Área Profissional da Saúde—Medicina Veterinária e Saúde, Faculdade de Ciências Agrárias e Veterinárias Jaboticabal Brazil

**Keywords:** Bland–Altman analysis, dog, method agreement, reagent strips, urinalysis, urinary pH, urine specific gravity

## Abstract

**Background:**

Urinalysis is a simple and essential test in veterinary practice, involving physical, chemical, and microscopic analysis of urine. Reagent strips are widely used due to their practicality; however, their reliability compared with reference laboratory methods remains a subject of debate.

**Objectives:**

This study compares different brands of reagent strips for the measurement of urinary pH and urine specific gravity (USG) in dogs and to evaluate their performance in relation to reference methods.

**Methods:**

This study evaluated the reliability of six brands of reagent strips for measuring pH and urine specific gravity in dogs, comparing them with the gold standard methods (bench pH meter and refractometer). Association between methods was evaluated using Pearson and Spearman correlation analyses, and agreement was assessed using Bland–Altman analysis. Linear and polynomial regression models were applied to derive correction equations where appropriate. Ninety‐seven canine urine samples were analyzed, collected by natural urination, and processed under refrigeration for preservation.

**Results:**

For pH analysis, reagent strips showed statistically significant associations with the benchtop pH meter, and Bland–Altman analysis demonstrated acceptable agreement, with small mean bias and relatively narrow limits of agreement. The linear and polynomial regression models generated to correct the strip values showed excellent fit, with coefficients of determination (*R* ^2^) greater than 69%, validating their use to calibrate results and improve accuracy. In contrast, urine specific gravity measurements obtained by reagent strips showed poor agreement with refractometry, characterized by wide limits of agreement and clinically relevant bias.

**Conclusions:**

The reagent strips are inadequate for reliable determination of urine specific gravity in dogs and are not recommended as a substitute for a refractometer, while showing acceptable agreement for urinary pH estimation. According to the proposal, its use is feasible in routine clinical practice, provided that the values are adjusted by the proposed correction equations, which minimize scaling errors and significantly improve the accuracy of the results, if the manufacturers' guidelines are followed.

## 1 Introduction

A urine test or urinalysis is a simple, low‐cost, and minimally invasive test for animals. It is used as a complementary test in veterinary clinics. The first stage of the test involves observing the urine in a transparent glass jar to check its volume, density, odor, and appearance (color and cloudiness). The second stage is a semi‐quantitative chemical test, using reagent strips to assess pH, protein, occult blood (hemoglobin), glucose, ketones, urobilinogen, nitrate, and leukocytes. The third step is the analysis of urinary sediment, with samples being centrifuged at 1500 rpm for 5 min, and microscopic quantification on a slide and coverslip [1, 2].

The method of urine collection can directly affect the results of the analysis. A standardized volume of urine must be obtained to enable comparison of the urinary sediment with subsequent samples. When collecting urine through natural urination, the first few streams of urine should be avoided, as they contain cells, bacteria, and debris from the urethra and vulva or foreskin, which can make the sample unrepresentative. For catheterization, there are specific catheters for each species and sex. A sterile lubricant will be applied to the tip of the catheter, which will then be inserted into the urethra and advanced to the urinary bladder. Due attention will be paid to the ease with which the catheter is inserted, as this can provide useful information about animals with dysuria. In this type of collection, proteinuria, hematuria, and the presence of a moderate number of cells are expected to be found in the urinalysis due to the collection method [3].

After collecting, it is recommended that urine analysis be performed within 30 min; otherwise, it should be refrigerated (1°C–4°C). Urine left at room temperature without refrigeration may undergo artifactual changes, including bacterial overgrowth, color change, deterioration of casts and epithelial cells, decreased concentrations of bilirubin, glucose, and ketones, dissolution and formation of crystals dependent on urine type, pH, and storage temperature. There may also be an increase in odor, turbidity, and pH. If the urine is alkaline and stored at room temperature, bacterial degradation may occur, or carbon dioxide loss may transform urea into ammonia, thereby altering the values of casts, ketone bodies, glucose, bilirubin, and epithelial cell integrity [4, 5]. Therefore, if immediate testing is not possible, the sample should be refrigerated at 4°C for a maximum of 12 h and then returned to room temperature (20°C–25°C) before re‐evaluation, as temperature changes may interfere with the enzymatic reaction on the reagent strips [6].

In general, several conditions must be observed to prevent inaccurate readings with reagent strips: ensuring that a refrigerated urine sample returns to ambient temperature prior to testing; preventing urine contamination by disinfectants from skin preparation or pre‐collection cleaning; using non‐expired reagent strips; avoiding storage issues related to exposure to air or light; preventing cross‐transfer of reagents between studies during result interpretation; performing the test readings at the appropriate time; accounting for highly pigmented urine; and verifying strip accuracy through appropriate urinary monitoring [7].

One way to assess urine specific gravity (USG) and pH in real time is to use reagent strips, which are readily available on the market, with the aim of facilitating, reducing the cost, and optimizing the time required for an examination. This urine reagent strip is a multi‐layer strip of paper and plastic, containing several pads impregnated with specific chemical reagents that react in the presence of urinary constituents. The interpretation of the result can be qualitative, with positive or negative outcomes, or semi‐quantitative, based on color changes corresponding to the concentration of these constituents [8]. With just one strip it is possible to measure pH, proteins, glucose, ketones, hemoglobin, bilirubin, urobilinogen, nitrite, urinary concentration, and leukocytes, making it possible to perform tests in less time [9, 10].

Urine Concentration is an especially useful indicator of renal urinary concentration ability, which reflects the kidney's ability to concentrate or dilute urine. It is defined as the ratio between the mass of a liquid volume and the mass of the same volume of distilled water. USG can be obtained by refractometry, which is the most commonly used method in clinical practice. Studies show that density is not influenced by the sample's storage time but can be affected by temperature. Therefore, when stored under refrigeration, the sample should be brought to ambient temperature (20°C–25°C) at the time of USG measurement [6, 9, 10].

The use of reagent strips to determine USG has not yet been adequately validated for use in veterinary medicine; however, several products of this type are available for sale on the market. Because the strips still provoke mistrust in laboratories, refractometry remains considered the standard procedure. USG readings with reagent strips are not yet reliable for dogs and cats and should be re‐evaluated by refractometers [11, 12]. Therefore, in this study, the values obtained by the reagent strip method and refractometry will be compared to better adjust the reagent strips.

Urine analysis is a great ally for accurate laboratory diagnosis. A good clinician needs to assess all parameters to determine the diagnosis. Thus, the use of complementary tests has become indispensable in routine veterinary practice. The objective of this study was to compare distinct brands of reagent strips to compare and correlate the pH and USG values obtained with the gold standard methods, refractometer, and bench pH meter used in laboratories. Based on this analysis, a comparison was made, generating a correction equation for the reagent strips.

## 2 Materials and Methods

The present study was conducted at the Clinical Pathology Laboratory of the Veterinary Hospital of the University of Brazil, Fernandópolis Campus, São Paulo, Brazil. The materials and methods of this study were approved by the Animal Research Ethics Committee of the University of Brazil under protocol number 1900025.

### 2.1 Sample Collection and Storage

The samples were collected in two veterinary clinical pathology laboratories (Clinical Pathology Laboratory of the Veterinary Hospital of Brazil University, Fernandópolis Campus, São Paulo, Brazil; TECSA CIAPAV Laboratory, located in São José do Rio Preto, São Paulo, Brazil) all through natural urination, using a sterile infant urine collector intended for human use but adapted for use in dogs. The samples were collected and stored refrigerated (at 4°C). Before being analyzed, they were left at room temperature (27°C) and homogenized.

### 2.2 Materials Used for pH and USG Testing

The same urine sample was analyzed by both reagent strips and reference methods, both for human and veterinary use (but all widely used in routine veterinary clinical analysis laboratories): Combur 10 Test M (RocheTM); Urofita 10DL (Alere); BIO 10 (Biocon); Uri—Color Check (Wama Diagnóstica); Uriquest Plus I (Labtest); Uriquest Plus VET (Labtest). During the use of the strips, the following were also used: disposable dropper pipette, glass test tubes (15 mL), distilled water, refractometer (Instrutemp Refractometer, Model ITREF‐200, São Paulo, Brazil), and digital bench pH meter (Hanna multiparameter photometer, Model HI83303‐02, Smithfield, Rhode Island, USA).

### 2.3 USG Analysis

In the refractometer analysis, a drop of urine was deposited onto the prism of the equipment, in the area indicated by the manufacturer, followed by its direct optical evaluation. We emphasize that before each analysis, the refractometer was calibrated with distilled water, whose specific gravity is equivalent to 1.000, ensuring measurement accuracy. When the specific gravity exceeded the maximum reading limit of the refractometer, the sample was diluted in a 1:1 ratio (one drop of urine to one drop of distilled water). After a new refractometric reading, the last two digits of the value obtained were multiplied by two, according to the methodology described for samples with high urinary concentration [13].

For strip analyses, these have specific squares for each item to be evaluated. In the USG item square, this was soaked with urine (20 μL/square) for reaction, and respecting the reaction time indicated by the manufacturer. After wetting, the strip was positioned laterally to allow drainage of excess urine by gravity, avoiding accumulation of liquid on the reagent fields. Throughout the procedure, the strip was kept in a horizontal position to prevent capillary migration between adjacent fields and minimize the risk of cross‐contamination. Reading was conducted as recommended by the strip manufacturers, according to the color obtained. The interpretation of the color was performed independently by two previously trained evaluators, without communication between them at the time of reading, to reduce observer bias and interobserver variability. In cases of disagreement, the consensus value was adopted after joint re‐evaluation [14]. Measurements obtained by reagent strips and refractometry were treated as paired observations for subsequent comparative analyses.

### 2.4 Urinary pH Analysis

Reagent strips with a pH indicator were used to determine urinary pH. These strips use a dual indicator system of methyl red and bromothymol blue to identify pH values between 5 and 9, producing a color change according to the alkalinity or acidity of the urine.

The reagent strips were soaked with the urine sample in the square indicated for pH analysis using a disposable dropper pipette; they were then left to stand for further analysis, in accordance with the manufacturer's instructions. The color obtained was compared with the standard color chart on the bottle, assigning the result to the corresponding pH value [15, 16].

After using the strips, the second urinary pH measurement was obtained using the digital bench pH meter. Daily, before each reading, the pH meter was calibrated with standard buffer solutions provided by the manufacturer, within the desired range (4–7 or 7–10), always starting with pH 7. To do this, the electrode was immersed in standard solution 7, taking care to completely cover its tip until the standard result was fully read and confirmed. After cleaning the electrode with deionized water and removing excess moisture with absorbent paper, the second buffer was read, repeating the same procedure as before. Finally, after a second cleaning with deionized water, the device was ready for urinary pH readings.

For each reading, the electrode was immersed directly in the urine sample, the device was allowed to stabilize, and the final values were recorded. The results were expressed as absolute numbers with two decimal places.

### 2.5 Statistical Analysis

The USG and pH values obtained by reagent strips and by reference methods (refractometer and benchtop pH meter) were analyzed to assess the relationship and agreement between methods. The PROC REG tool from SAS software (SAS Institute, Cary, NC, USA) version 9.4, 2014. Thus, the Linear Equation *Y* = *a* + *bX* and the Polynomial Equation *Y* = *a* + *bx* + *cx* ^2^ were applied to adjust the relationship between *Y*, the estimated component of the reagent strips, and *X*, the component estimated by the refractometer and pH meter.

The equations used have two or more input variables to model the relationship between methods and derive correction equations for USG and urine pH, according to linear and polynomial regression.

The results for USG and pH determined by refractometer and pH meter analyses were compared to the measurements estimated by the test strip under study, with model fit evaluated using the coefficient of determination (*R* ^2^), which is a statistical measure of how close the data are to the adjusted regression line.

Agreement between reagent strips and reference methods was evaluated using Bland–Altman analysis, with calculation of mean bias and 95% limits of agreement (±1.96 standard deviations). Differences between paired measurements were plotted against their mean values to assess systematic bias and clinical agreement.

## 3 Results

Reagent strips for detecting pH and USG are widely used, but they are less accurate than pH meters and refractometers [17]. Accordingly, measurements obtained by the bench pH meter and refractometer (reference methods) were compared with those obtained by reagent strips to assess method performance.

Table 1 summarizes the mean ± standard deviation for urinary pH and USG measured by the reference methods (benchtop pH meter and refractometer) and by the reagent strips. To evaluate the values found by standard pH and USG methods (pH meter and refractometer) in relation to reagent strips, Pearson and Spearman correlation analyses were performed (Table 2). Correlation analyses were used to quantify the strength of association between methods. Agreement between methods was assessed using Bland–Altman analysis. Pearson's correlation was used for linear association and Spearman's correlation for monotonic association (Table 2).

**TABLE 1:** Average values for pH and urine specific gravity, evaluated using standard methods and reagent strips.

	*N*	Average	Standard deviation
pH assessment			
pH meter	97	6.67	1.05
DUS 10	97	6.36	0.68
Uriquest Plus VET	97	6.08	0.90
Urofita 10DL	97	6.46	0.87
Uriquest Plus I	97	6.19	1.01
BIO 10	97	6.33	0.58
Combur 10 Test M	97	6.45	0.95
Uri‐Color Check	97	6.30	0.88
USG assessment			
Refractometer	97	1026.82	17.29
DUS 10	97	1025.77	6.31
Urofita 10DL	97	1018.09	7.52
Uriquest Plus I	97	1022.78	7.29
BIO 10	97	1021.75	6.92
Combur 10 Test M	97	1017.58	6.58
Uri‐Color Check	97	1019.07	6.31

*Note: N*—samples.

**TABLE 2:** Correlation values for pH and urine specific gravity, evaluated by standard methods and reagent strips.

Strips	pH test			Density test	
	Correlation		*p*	Correlation	*p*
DUS 10	Pearson	0.680	0.0001	−0.106	0.303
	Sperman	0.799	0.0001	−0.057	0.581
Uriquest Plus VET	Pearson	0.643	0.0001	0.145	0.157
	Sperman	0.824	0.0001	0.264	0.009
Urofita 10DL	Pearson	0.669	0.0001	0.140	0.171
	Sperman	0.841	0.0001	0.226	0.026
Uriquest Plus I	Pearson	0.692	0.0001	—	—
	Sperman	0.868	0.0001	—	—
BIO 10	Pearson	0.668	0.0001	0.131	0.200
	Sperman	0.780	0.0001	0.213	0.036
Combur 10 Test M	Pearson	0.684	0.0001	0.052	0.612
	Sperman	0.861	0.0001	0.222	0.029
Uri‐Color Check	Pearson	0.400	0.0001	0.184	0.071
	Sperman	0.781	0.0001	0.345	0.001

Table 2 shows that the correlations between the standard method and the pH reagent strips were all significant (*p* < 0.05), indicating a statistically significant association. For Pearson's correlation, the Uriquest Plus I strip had the highest correlation value (0.692), while the Uri‐Color Check strip had the lowest value (0.400). For Spearman's correlation, all reagent strips showed significant associations (*p* < 0.05) (Figure 1) showed significant associations.

**FIGURE 1:**
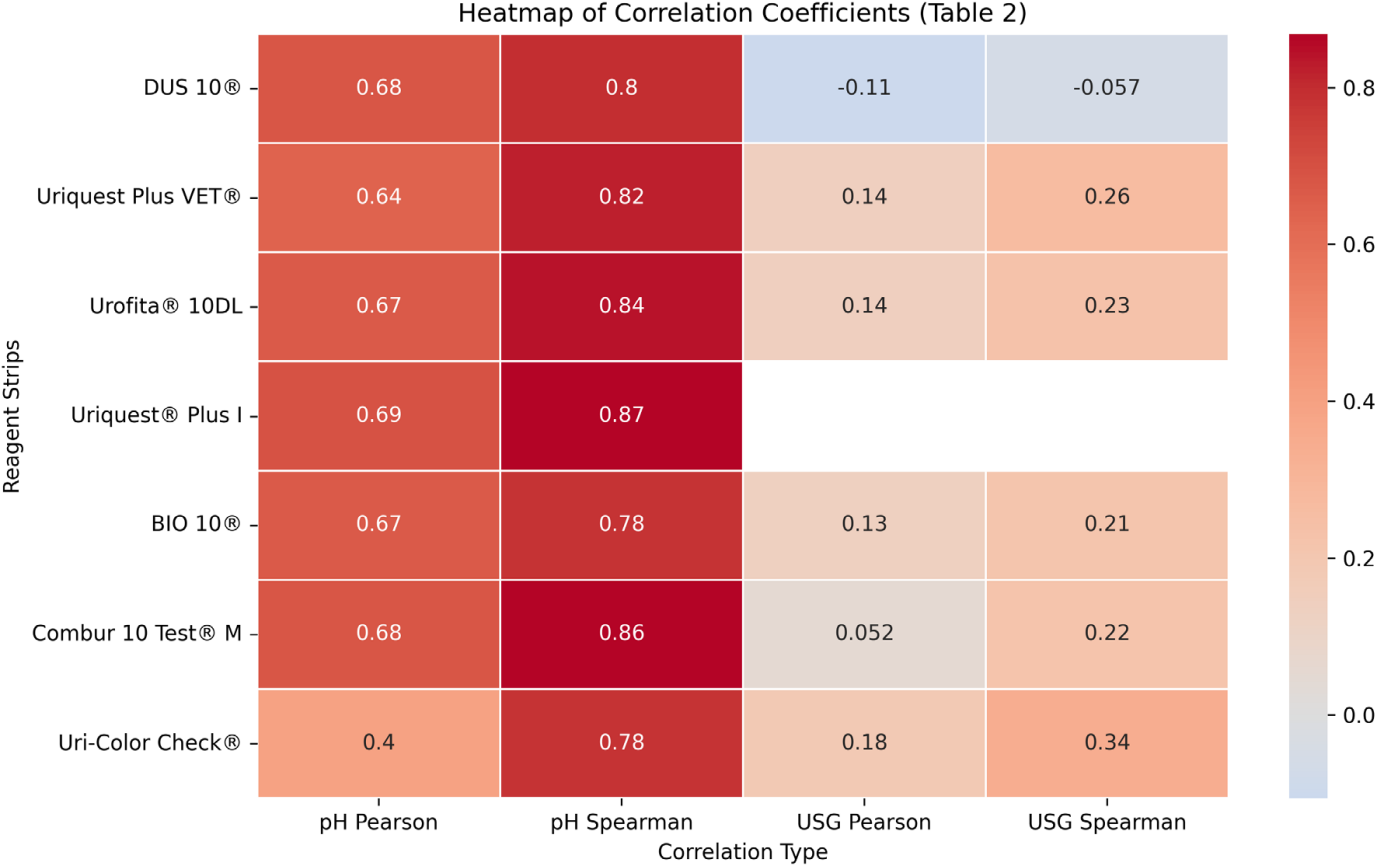
Heatmap of correlation coefficients between reagent strips and gold standard methods for urinary pH and urine specific gravity (USG) in canine samples. Colors represent the magnitude and direction of Pearson and Spearman correlation coefficients, with warmer colors indicating stronger positive correlations and cooler colors indicating weak or negative correlations.

Agreement between reagent strips and the benchtop pH meter was further evaluated using Bland–Altman analysis (Figure 2). Overall, the plots demonstrated a small mean bias across all reagent strips, with most differences distributed symmetrically around zero across the range of measured pH values. The limits of agreement were relatively narrow and consistent among strips, indicating acceptable agreement with the reference method. No clear trend toward proportional bias was observed, indicating no evident proportional bias across the pH range (Figure 2).

**FIGURE 2:**
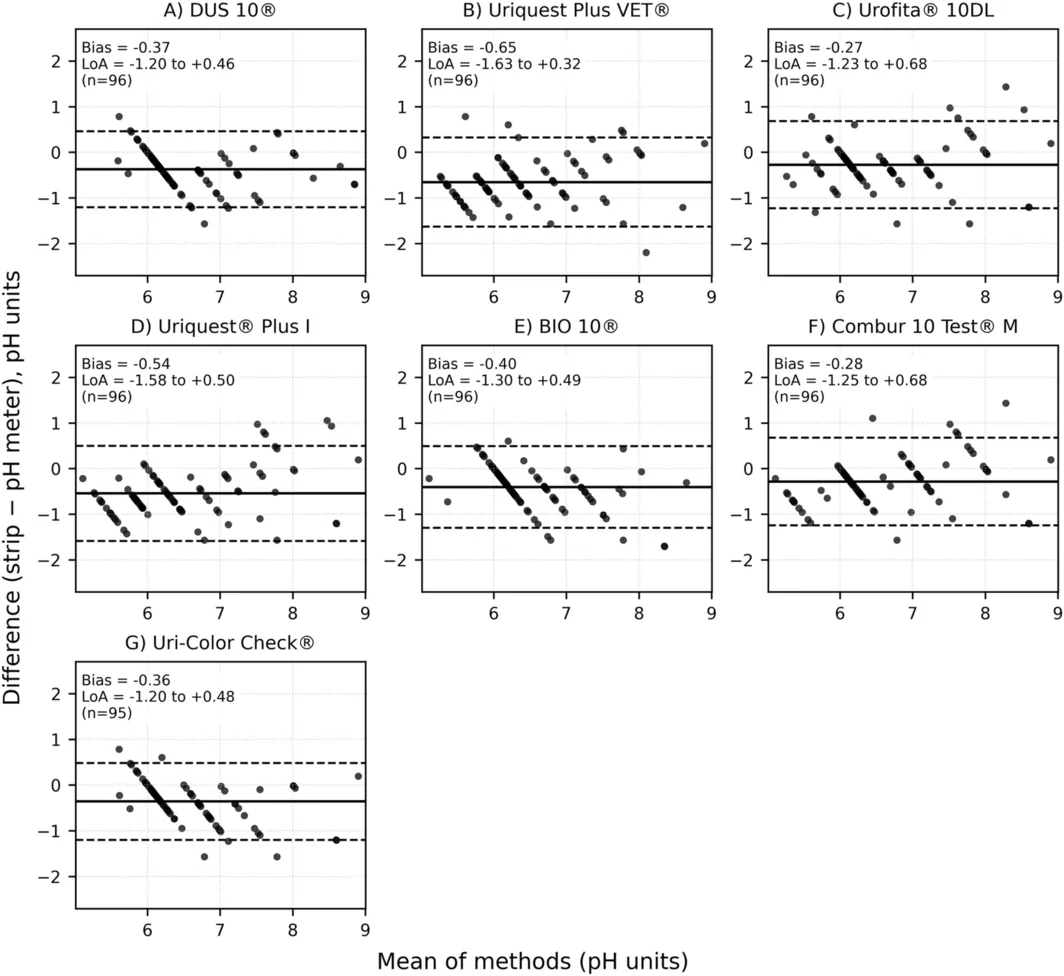
Bland–Altman agreement plots for urinary pH measured by reagent strips versus the benchtop pH meter (reference method). Each panel (A–G) shows the difference between methods (strip—pH meter) plotted against the mean of the two methods. Solid lines indicate the mean bias, and dashed lines indicate the 95% limits of agreement (bias ±1.96 SD).

For USG association, the reagent strips showed low values for Pearson correlation, with only the Uri‐Color Check strip showing significance (*p* < 0.05), albeit with a low value (0.184). For Spearman's correlation, only the DUS 10 reagent strip did not show significance, indicating no statistically significant monotonic association with refractometry. Although the Uriquest Plus VET, Urofita 10DL, BIO 10, Combur 10 Test M, and Uri‐Color Check strips showed significant correlations for the Spearman test, the values were low, indicating that the values tend to change simultaneously, but not necessarily at a constant rate, indicating that there may be inconsistency in the association pattern across strips (Figure 1).

Bland–Altman analysis revealed poor agreement between reagent strips and refractometry for urine specific gravity measurements (Figure 3). The plots showed wide limits of agreement and substantial dispersion of differences across the entire range of USG values. Several reagent strips exhibited systematic bias, with differences that were not randomly distributed around zero. These findings indicate clinically relevant disagreement between methods, reinforcing that reagent strips are not reliable substitutes for refractometry in the assessment of urine specific gravity in dogs. Overall, the magnitude of disagreement was considered clinically unacceptable for substituting refractometry.

**FIGURE 3:**
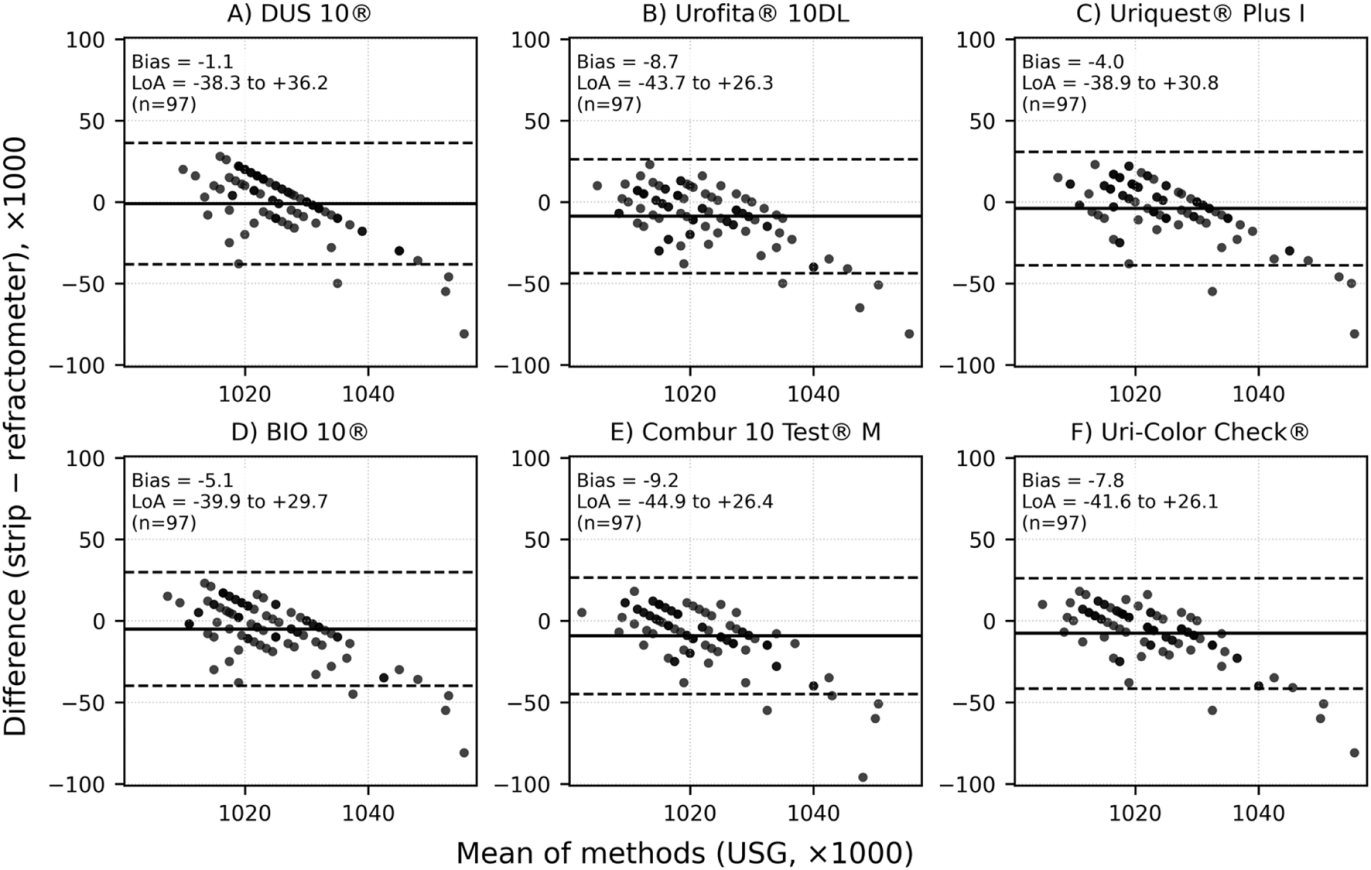
Bland–Altman agreement plots for urine specific gravity (USG) measured by reagent strips versus refractometry (reference method). Each panel (A–F) shows the difference between methods (strip—refractometer) plotted against the mean of the two methods (USG expressed as specific gravity ×1000). Solid lines indicate the mean bias, and dashed lines indicate the 95% limits of agreement (bias ±1.96 SD).

Regression models were then fitted to derive correction equations for reagent strip measurements in relation to the reference methods (Table 3; Figures 2, 3, 4).

**TABLE 3:** Regression equation for pH and urine specific gravity, evaluated by standard methods and reagent strips.

Strips	Models	Equations for pH	*R* ^2^
DUS 10	Linear	pH = 0.3726 + 0.9994 × Dus	0.7215
	Polynomial	pH = −7.736 + 3.364 × Dus − 0.1695 × Dus^2^	0.7264
Uriquest Plus VET	Linear	pH = 2.215 + 0.7424 × Uri	0.6985
	Polynomial	pH = 1.661 + 0.9173 × Uri − 0.01348 × Uri^2^	0.6988
Urofita 10DL	Linear	pH = 1.762 + 0.7693 × Uro	0.6984
	Polynomial	pH = −2.253 + 1.949 × Uro − 0.08490 × Uro^2^	0.7085
Uriquest Plus I	Linear	pH = 2.525 + 0.6800 × Urp	0.7293
	Polynomial	pH = −0.813 + 1.705 × Urp − 0.07644 × Urp^2^	0.7417
BIO 10	Linear	pH = −0.5466 + 1.149 × Bio	0.6900
	Polynomial	pH = −3.778 + 2.125 × Bio − 0.07295 × Bio^2^	0.6924
Combur 10 Test M	Linear	pH = 2.072 + 0.7225 Com	0.7376
	Polynomial	pH = 1.925 + 0.7669 × Com − 0.00330 × Com^2^	0.7377
Uri‐Color Check	Linear	pH = −0.2217 + 1.089 × Urc	0.6985
	Polynomial	pH = −11.82 + 4.475 × Urc − 0.2435 × Urc^2^	0.6988

Strips	Models	Equations for urine specific gravity	*R* ^2^
Uri‐Color Check	Linear	Ref = 513 + 0.5042 × Urc	0.0338
	Polynomial	Ref = 62 414 + 121.3 × Urc − 0.05987 × Urc^2^	0.0691

**FIGURE 4:**
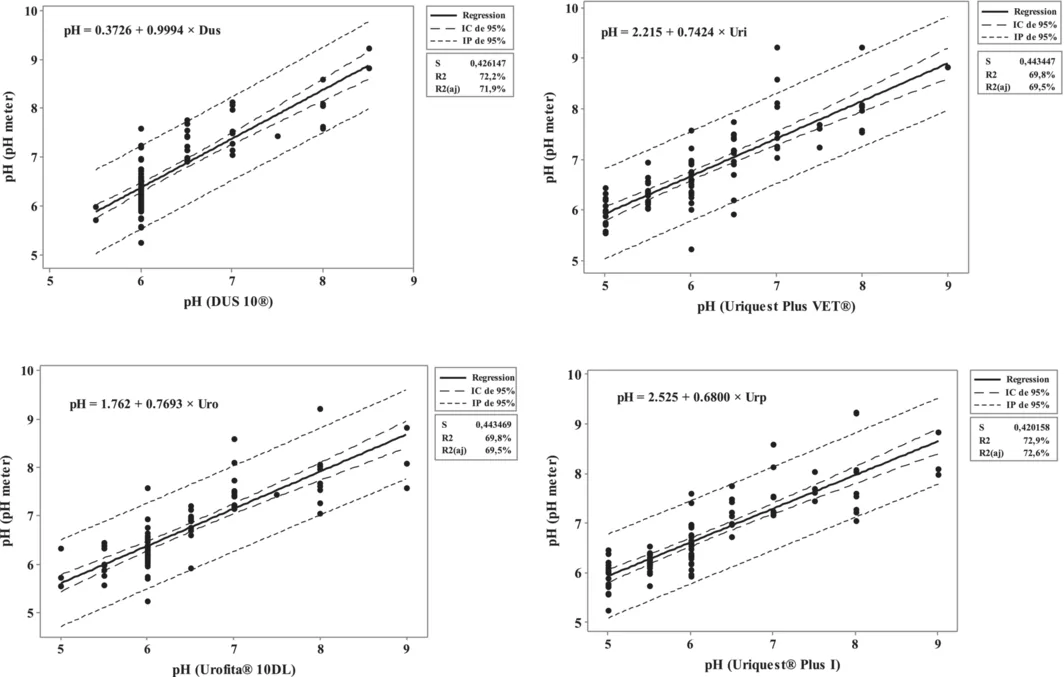
Linear correlation between urinary pH values obtained by the gold standard method (benchtop pH meter) and by reagent strips (DUS 10; Uriquest Plus VET; Urofita 10DL; Uriquest Plus I) in canine samples. The regression line, the coefficient of determination (*R* ^2^), and the standard error (S) were calculated. The confidence intervals (95% CI) and prediction (95% PI) represent, respectively, the precision of the mean fit line and the expected range for new individual variations.

Based on the presented results, the linear model was selected as the best choice for adjusting the regressions aimed at correcting the pH values obtained by the reagent strips, due to its combination of simplicity, efficacy, and high predictive power. Initially, the significant correlations (*p* < 0.05) and the strong Spearman correlation coefficients (ranging from 0.781 to 0.868) between all strips and the benchtop pH meter (gold standard) justified the application of regression, confirming a robust monotonic relationship. When adjusting the models, both simple linear regression (*Y* = *a* + *bX*) and quadratic polynomial regression (*Y* = *a* + *bx* + *cx* ^2^) demonstrated excellent goodness‐of‐fit, with coefficients of determination (*R* ^2^) greater than 69% for all evaluated brands (Table 3). Although the polynomial models showed slightly higher *R* ^2^ values in some cases, the explanatory gain was marginal compared to the linear model, which proved to be parsimonious and sufficient to capture the relationship between the methods (Figures 1, 2, 3, 4). The high magnitude of the *R* ^2^ in the linear model validates its use to calibrate the strip results, allowing for the correction of systematic scale errors and significantly improving the accuracy of pH estimation in relation to the reference method, as proposed in the study. As for USG regression, it was applied only to the Uri‐Color Check strip; however, it yielded a very low adjustment value (> 3%), which may cause problems when correcting the strip value relative to the gold standard method (refractometer).

## 4 Discussion

Urinalysis is a routine test used in small animal clinics and is extremely helpful in obtaining diagnoses and conducting treatment and follow‐up of patients [18, 19].

Reagent strips, widely used for urinalysis in veterinary medicine, provide information on the function of the urinary system, but other organs can also be evaluated, such as the endocrine pancreas and liver. It also provides preliminary information regarding glomerular hemorrhage, liver disease, and urinary tract infections through indicators such as pH and urine specific gravity [20]. However, their clinical usefulness depends on the level of agreement with reference methods (Figures 5 and 6).

**FIGURE 5:**
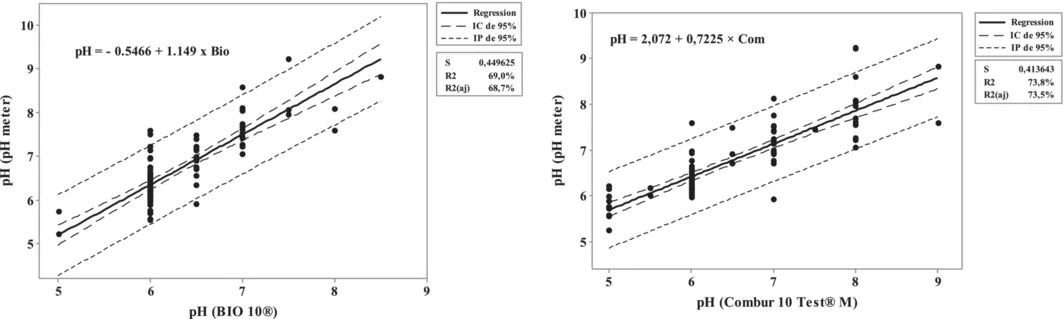
Linear correlation between urinary pH values obtained by the gold standard method (benchtop pH meter) and by reagent strips (BIO 10; Combur 10 Test M) in canine samples. The regression line, the coefficient of determination (*R* ^2^) and the standard error (S) were calculated. The confidence intervals (95% CI) and the prediction intervals (95% PI) represent, respectively, the precision of the mean fit line and the expected range for new individual measurements.

**FIGURE 6:**
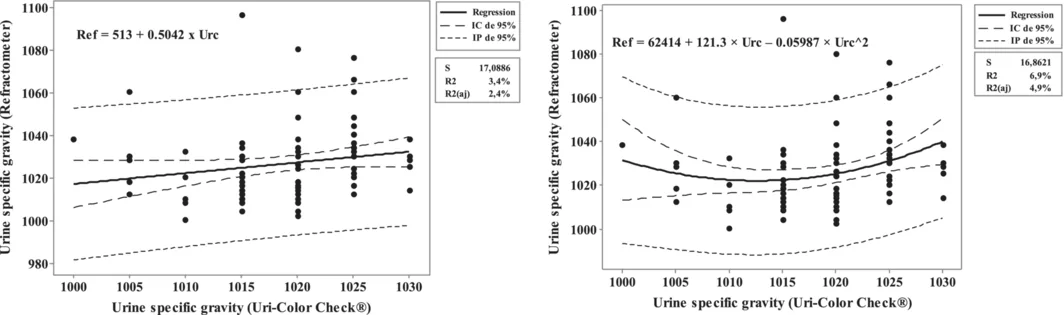
Linear and polynomial correlation between urine specific gravity values obtained by the gold‐standard method (benchtop pH meter) and by reagent strips (Uri‐Color Check) in canine samples. The regression line, the coefficient of determination (*R* ^2^) and the standard error (S) were calculated. The confidence intervals (95% CI) and prediction intervals (95% PI) represent, respectively, the precision of the mean fit line and the expected range for new individual measurements.

The pH of urine is determined by H+ ions, which vary according to each animal's metabolism. In carnivores, urine is normally acidic; in herbivores, alkaline. USG is a particularly useful test for assessing renal dilution and concentration capacity, as loss of concentration ability is usually the first sign of renal tubular disease, and consequently lower urinary concentration. The USG value in healthy patients may vary according to body hydration and fluid consumption, and is related to glomerular pathologies and may be suggestive of or confirm several other pathologies [21].

During the study period, urine from 97 animals of different ages and health status was used. For urinary pH analysis, the average found by the pH meter was higher than that found by the strips, reflecting systematic differences between methods, as well as a higher standard deviation, demonstrating a wider reading range by the standard method. Bland–Altman analysis further demonstrated acceptable agreement between reagent strips and the pH meter, supporting their use for approximate urinary pH assessment. Urine pH can be directly influenced by diet, such as a recent meal, meat consumption, or low‐protein diets; by disease states affecting the body's acid–base balance; by bacterial infection; by chronic vomiting and/or diarrhea; by pyrexia; by protein catabolic states; by renal tubular acidosis; by certain drugs (ascorbic acid, bicarbonate, and potassium citrate); and by storage time prior to processing [11, 22]. The urine of carnivores such as dogs and cats is normally acidic, with reference values varying according to some authors: between 6 and 7.5 [23] or between 5 and 7.5 [24].

The USG values (Table 1) obtained by the standard method (refractometer) and by pH were also higher than those obtained with the strips, and the standard deviation was higher, indicating a wider range than with the strips. This wider dispersion was consistent with the wide limits of agreement observed in the Bland–Altman analysis. Urinary concentration depends on the number, size, and molecular weight of the particles present in the solution, as it represents the ratio between the refractive index of urine and that of distilled water. Although osmolarity provides a more accurate value of urinary concentration than USG, it is easier to measure [25]. Under normal conditions, urine is composed of low molecular weight particles, so urinary concentration does not differ from osmolarity. On the other hand, if urine contains high‐molecular‐weight substances (glucose or mannitol), these substances will have a much greater effect on urinary concentration than on osmolarity [24].

Studies have shown that pH measurements using strips are inaccurate compared to those using a pH meter. Therefore, a properly calibrated pH meter should be used when accurate pH values are required for clinical decision‐making [23]. Urine strips estimate pH at one unit above or below the value measured by the pH meter. In cats, this estimate is underestimated, and in dogs, there is no consistency in the direction of the error [26]. In a study comparing the measurement of feline urine pH using a pH meter with urine strips, it was concluded that urine strips are useful when an approximation of pH is desired, such as in routine urine analysis, but should not be used when accurate pH monitoring is required [27].

USG can also be calculated using urine strips by measuring the color change of a pH indicator, bromothymol blue, but this is inaccurate in veterinary medicine and is not recommended [24]. The present agreement analysis corroborates these findings. A refractometer is the best technique for measuring specific gravity in clinical practice. Several authors have compared the osmolarity of dog urine with the specific gravity obtained with a refractometer and urine test strips, concluding that a refractometer should be used when it is not possible to calculate osmolarity and that urine test strips should never be used to measure specific gravity [5]. It should be remembered that density should be measured before initiating treatment, since fluid therapy, diuretics, and glucocorticoids can alter the results, thus causing the results to fall outside the standard established by the strips, with the refractometer being indicated in these cases [24].

The best adjustment values found for pH reagent strips can be explained by the material they use. The reagents used in pH measurement strips include methyl red, bromothymol blue, and phenolphthalein, all of which can detect pH values between 5 and 9 [28].

One possible explanation for poor agreement between reagent strips and refractometry for USG lies in the chemical composition of canine urine, which differs substantially from human urine for which many strips were originally designed. Factors such as diet, collection timing, and the presence of interfering substances may further compromise strip performance [29]. Studies indicate that even chemical test strips manufactured for the evaluation of canine and feline urine are not recommended for assessing specific gravity, urobilinogen, nitrite, or leukocytes [5, 11, 12, 22].

This study is in line with other studies in the literature [19], who reported that it is essential to standardize the aspects involved in this type of test, as well as the information contained in the package inserts regarding the definition of actual interferences and the concentrations at which they pose a risk to the interpretation of the results. It is also necessary to standardize the legends and symbols used, allowing for equal interpretation of the results regardless of the location, analyst, or brands of reagents used in the analysis, thus avoiding erroneous diagnoses, especially regarding the importance of validation based on agreement rather than exclusive reliance on measures of association.

Given the difficulty of using reagent strips for certain parameters, such as specific gravity, it is important for veterinarians to employ the strips as screening tests, always correlating the results with the patient's clinical findings and suspected conditions. In cases of inconsistencies, the results should be correlated with reference laboratory methods [30].

## 5 Conclusions

Based on agreement and regression analyses, it can be concluded that the reagent strips used in human and veterinary medicine evaluated in this study for measuring urinary pH can be used with acceptable clinical agreement as demonstrated by Bland–Altman analysis, provided that the correct recommendations and standards are followed. The regression models generated based on the gold standard method (pH meter) and the test strips can reduce scale errors in the evaluation when applied as correction equations. It was also possible to verify that the use of reagent strips in dog urine, especially in relation to the USG parameter, is not recommended, presenting a poor clinical agreement and should not be considered a substitute for the reference method in dogs. These findings reinforce the need for improved standardization of reagent strip reactions and clearer information in package inserts to minimize misinterpretation of results in clinical practice.

## Conflicts of Interest

The authors declare no conflicts of interest.

## Data Availability

The data that support the findings of this study are available from the corresponding author upon reasonable request.
